# Short-Term Local Adaptation of Historical Common Bean (*Phaseolus vulgaris* L.) Varieties and Implications for In Situ Management of Bean Diversity

**DOI:** 10.3390/ijms18030493

**Published:** 2017-02-28

**Authors:** Stephanie M. Klaedtke, Leonardo Caproni, Julia Klauck, Paul de la Grandville, Martin Dutartre, Pierre M. Stassart, Véronique Chable, Valeria Negri, Lorenzo Raggi

**Affiliations:** 1 INRA–Agrocampus Ouest-ESA Angers, UMR 980 BAGAP, 65 rue de St. Brieuc, CS 84215, 35042 Rennes, France; stephanie.klaedtke@inra.fr (S.M.K.); klauckjulia@yahoo.de (J.K.); Paul.DeLaChevardiereDeLaGrandville@inra.fr (P.d.l.G.); martin.dutartre@supagro.fr (M.D.); Veronique.Chable@inra.fr (V.C.); 2 Socio-Économie, Environnement et Développement, Département des Sciences et Gestion de l’Environnement, Liège University, Av. Longwy 185, 6700 Arlon, Belgium; P.Stassart@ulg.ac.be; 3 Dipartimento di Scienze Agrarie, Alimentari e Ambientali (DSA3), Università degli Studi di Perugia, Borgo XX Giugno 74, 06121 Perugia, Italy; leonardo.caproni@hotmail.it (L.C.); valeria.negri@unipg.it (V.N.)

**Keywords:** common bean, local adaptation, genetic diversity, microevolution, Simple Sequence Repeat (SSR) markers, crop diversity, organic farming

## Abstract

Recognizing both the stakes of traditional European common bean diversity and the role farmers’ and gardeners’ networks play in maintaining this diversity, the present study examines the role that local adaptation plays for the management of common bean diversity in situ. To the purpose, four historical bean varieties and one modern control were multiplied on two organic farms for three growing seasons. The fifteen resulting populations, the initial ones and two populations of each variety obtained after the three years of multiplication, were then grown in a common garden. Twenty-two Simple Sequence Repeat (SSR) markers and 13 phenotypic traits were assessed. In total, 68.2% of tested markers were polymorphic and a total of 66 different alleles were identified. F_ST_ analysis showed that the genetic composition of two varieties multiplied in different environments changed. At the phenotypic level, differences were observed in flowering date and leaf length. Results indicate that three years of multiplication suffice for local adaptation to occur. The spatial dynamics of genetic and phenotypic bean diversity imply that the maintenance of diversity should be considered at the scale of the network, rather than individual farms and gardens. The microevolution of bean populations within networks of gardens and farms emerges as a research perspective.

## 1. Introduction

Common bean *(Phaseolus vulgaris* L.; 2*n* = 2*x* = 22), a prevalently autogamous species, is native of the New World and shows an Andean and a Mesoamerican gene pool, which are clearly distinct for both morphological [1] and genetic traits [2]. It was introduced into Europe after 1492. Upon its introduction onto the Iberian peninsula from the Americas, hybridization of the Andean and Mesoamerican gene pools created novel genetic variation [3,4,5,6] and Europe is considered a secondary center of diversity for common bean [7]. Europe’s landraces and historical varieties of this species constitute a cultural good and a promising resource for plant breeding [6,7]. In general, common bean is a highly variable species and uses are diverse. According to consumer preferences and bean varieties, either the pods are harvested as green beans or the mature seeds are harvested as dry beans. In Western Europe, the consumption of dry beans, historically considered as “meat for the poor”, has dropped over the last decades (1.3 kg per capita in 2013) [8]. In this geographic region, green beans, i.e., fresh pods, represent the main mode of consumption. To meet the demand for green beans all year round, the EU imported 196,700 t of fresh green bean in 2015, mainly from Morocco (64% of imports), Kenya (16%) and Egypt (10%) [9]. Nevertheless, green beans remain an important crop in the EU. France alone cultivated 26,830 ha in 2014, i.e., 28% of the surface harvested in the EU (97,280 ha), mainly for the canning and freezing industry [10]. Given a decreasing demand for dry beans on the one hand and the development of modern green bean varieties adapted to African growing environments and to large-scale mechanized agriculture in Europe on the other hand, traditional European bean diversity is at stake. Traditional European bean diversity, landraces and old varieties, show inter- and intra-population diversity [3,4,5,6,7] and were once largely cultivated.

Recently, the demand of organic market gardeners engaged in local food systems for crop diversity is increasing [11,12,13]. As for other vegetable crops, some home and market gardeners, especially organic producers, regard historical bean varieties and landraces as a promising alternative to modern varieties. Historical varieties are preferred for their agronomical traits, as they are often considered more tolerant to environmental stress factors and better suited for organic and low input growing conditions [11,13], but also for better taste and nutritional properties [13,14]. In this context, historical varieties are valued for their legal reproducibility, as they fall into the public domain and can be freely multiplied by farmers [11,12]. Home gardens and local organic food systems thus harbor potential for the use and maintenance of common bean diversity. Networks of both non-profit seed-saver associations and small-scale seed companies are engaged in the in situ management of European bean diversity by multiplying and disseminating bean seed of historical varieties [12]. This engagement pursues the dual aim of: (i) safeguarding crop diversity in gardens and fields; and (ii) offering home and market gardeners the opportunity to cultivate, use and multiply historical varieties and profit from their benefits. In this context, local adaptation of bean populations may contribute to both these aims. The underlying hypothesis is that, as plant populations adapt locally: (i) crop diversity evolves with environmental conditions; and (ii) morpho-phenological traits are shaped by environmental conditions and conscious selection. 

Improving our knowledge about both genetic and phenotypic diversity, as well as the possible evolution of plant populations in different environments and management systems over generations, appears to be crucial in order to understand local on-farm adaptation [15]. Since Morgante and Olivieri [16] have demonstrated the feasibility of using microsatellite markers (Simple Sequence Repeat (SSR)) in plants, several studies have been conducted on *P. vulgaris* and many SSR markers developed [17,18,19,20,21]. Microsatellites are single-locus, multi-allelic, codominant and highly reproducible markers. They therefore constitute an effective and reliable tool to dissect the level and structure of genetic diversity among bean populations [22]. Previous studies have employed microsatellites to assess and understand the microevolution of crop populations developed under different management systems including low-input and organic agriculture. This has been the case for cereal crops like wheat [15,23] and barley [24], but also legumes such as common bean [25,26]. At the phenotypic level, adaptation to local on-farm conditions has been revealed on crops such as lentil [27] and wheat [28,29]. 

In collaboration with an association of small-scale organic seed companies operating in France, Belgium and Luxembourg, the present research was conducted with the objective of assessing the adaptation of historical bean varieties (which can be referred to as heterogeneous varieties or populations, being composed of different varietal types or genotypes) to local growing environments, both at the genetic and at the phenotypic level. To the purpose, four historical bean varieties and a modern variety (used as control) were multiplied in two farm environments over three years. The fourth year, the resulting populations, as well as the original ones were grown in a common garden. Both morpho-phenological traits and genetic markers were assessed. Results confirm that most of the studied bean populations multiplied in the tested growing environments differentiate at both these levels within three years. Conclusions are drawn to support the in situ management of bean diversity in gardens and on farms.

## 2. Results

Three different populations for each one of the five studied bean varieties (total of 15 populations) were assessed by means of 22 SSR markers and 13 phenotypic traits. In particular, for each variety, the three populations were composed as follows: the initial one used to establish the multiplications (i.e., the original seed lot (ORI)) and two populations derived from each initial population by multiplying it in two contrasting growing environments (i.e., Brittany (BZH) and Luxembourg (LUX)) for three consecutive years and under organic management conditions. Hereafter, each population is designated by a code combining the abbreviation of the variety name and of the multiplication site/origin as reported in Table 1. 

DNA was successfully extracted from 470 individuals and subsequently SSR genotyping resulted in the production of about 20,000 data points. Among the 22 tested markers AG01, BM137, BM157, BM170, BM 172 and BM201 resulted monomorphic, while BMd-43 was excluded because of problems in the interpretation of the allelic pattern. A total of 66 different alleles were identified by means of the residual 15 polymorphic SSR markers (BM114, BM140, BM141, BM156, BM175, BM189, BM200, BMb221, BMb293, BMb356, BMb526, BMb619, BMd-41, BMd-44, and GATS91). Number of alleles per locus ranged from two (BM156, BMb293, and BMd-44) to eight (BM114, and BMb356) with a mean value of 4.4 alleles per polymorphic marker. 

**Figure 1:**
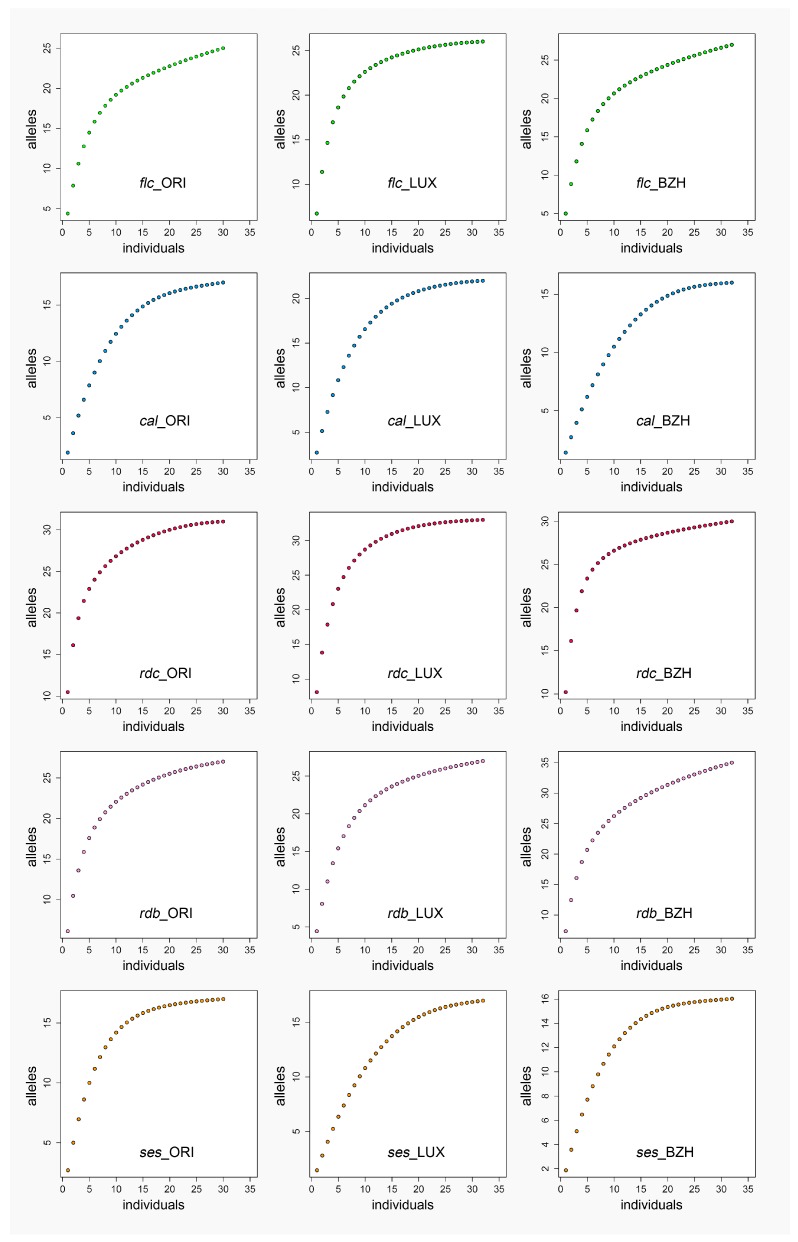
Mean SSR allelic richness accumulation curves of studied populations. Populations are coded according to Table 1.

Populations of the varieties *flc*, *rdc*, and *rdb* showed a higher mean number of alleles (from 26.0 to 31.3) when compared to *cal* and *ses* ones (18.3 and 16.3, respectively). The shape of mean SSR allelic richness accumulation curves showed that the highest proportion of alleles had been sampled for all populations and very few new alleles would have been discovered by increasing the number of analyzed individuals per population (Figure 1). Numbers of shared alleles among populations of the same variety ranged from 15 (*cal* and *ses*) to 25 (*rdc*) while the number of private alleles ranged from zero (*cal_BZH*) to nine (*rdb_BZH*). Venn diagrams showing the distribution of alleles among populations are reported in Figure S1. 

Morpho-phenological characterization was effectively carried out on 540 single plants grown in the common garden experiment. Among the assessed traits, days to flowering and leaf length showed strongest evidence for local adaptation, as most varieties were concerned.

### 2.1. Genetic Diversity

Analysis of Molecular Variance (AMOVA) carried out on the total dataset located 76%, 20% and 4% total diversity among populations, individuals and within individuals, respectively (not reported). Results of the same analysis, carried out separately for each variety, are reported in Table S1. Results showed that the highest differentiation among populations occurred in *flc* and *rdc* varieties (8.44% and 14.34% of total variation, respectively, *p* ≤ 0.001).

**Figure 2:**
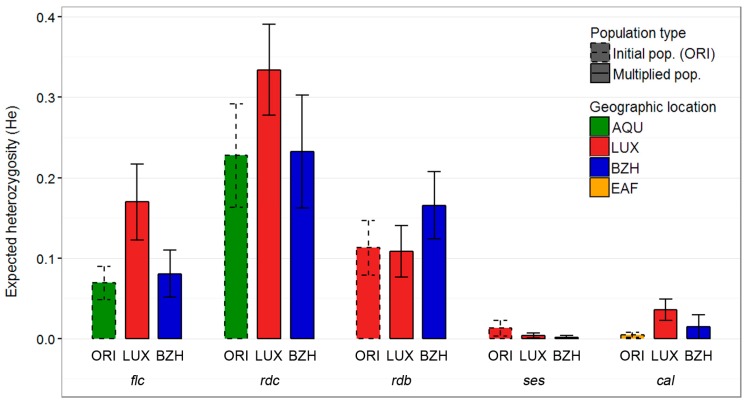
Expected heterozygosity (*He*) of three experimental populations of five common bean varieties, respectively. Colors of bars indicate the geographic location where seed lots were grown.

Expected heterozygosity (*He*) was computed as a measure of genetic diversity within populations (Figure 2). *He* values of the initial populations (ORI), which were used to establish the trial, ranged from 0.005 to 0.227, being the lowest for the modern control variety *cal* and the highest for *rdc*. Except for variety *ses*, within populations diversity showed a tendency to increase when the original seed lot was transferred to a new geographic location. By contrast, for the varieties *rdb* and *ses* diversity remained stable or decreased in the populations obtained after the three years of multiplication in the same environment from which the original seed lot had been obtained (LUX).

### 2.2. Genetic Differentiation

**Figure 3:**
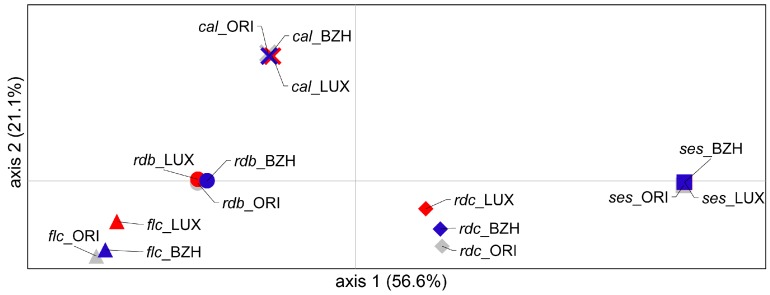
Two-dimensional plot of the principal coordinate analysis (PCoA) showing the clustering of the studied populations. Different symbols refer to different varieties. ORI populations are represented in grey, LUX in red and BZH in blue.

Principal coordinate analysis (PCoA) was performed to provide spatial representation of the relative genetic distances among populations. In total, 77.7% of diversity is explained by the first two axes (Figure 3): 56.6% by the first and 21.1% by the second. According to PCoA results, the populations clearly group according to the variety. Axis 1 separates the two large-seeded varieties *ses* and *rdc* from the other three varieties while Axis 2 distinguishes among the three smaller-seeded varieties, *flc*, *rdb* and *cal*. 

Whereas the three populations of the varieties *rdb*, *ses*, and *cal* almost completely overlap, suggesting a very low differentiation among them, the LUX populations of both *flc* and *rdc* demarcate from the BZH and ORI ones, indicating a clear differentiation.

To test for genetic differences among populations of a given variety, pairwise F_ST_ were also calculated. Results indicated that *flc_*LUX and *rdc_*LUX differed significantly from the respective original population (ORI) and the ones multiplied in BZH (*p* ≤ 0.001). In particular, among significant comparisons, a moderate differentiation was observed for *flc* populations while the differentiation was high among *rdc* populations. No significant differences were observed in any of the other pairwise comparisons (Table 2).

### 2.3. Population Genetic Structure Analysis

**Figure 4:**
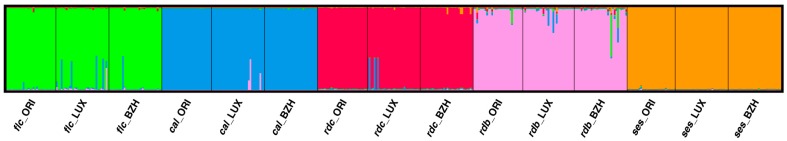
Genetic relationships among the 15 populations of common bean estimated using InStruct software. Each individual is represented by a vertical column divided into *K* colored segments. The length of each segment indicates the proportions of the genome attributed to the different clusters.

The Evanno test on STRUCURE results clearly indicated *K* = 5 as the most likely level of population subdivision consistently with the number of studied varieties (Figure S2). A graphic representation of population structure obtained by the InStruct software (*K* = 5) is reported in Figure 4. The results clearly grouped the individuals according to the variety while no differences among populations of the same variety was observed (Figure 4). 

Despite conscious selection of seeds during the three multiplication cycles, which consisted of removing off-type seeds from seed lots, some admixture of the populations multiplied in BZH and LUX was observed in all tested varieties, with the only exception of *ses*. When compared with the other populations, the presence of admixed individuals is particularly relevant in *flc_*LUX (15.6%) and in *rdc_*LUX (9.4%) while populations *cal_*LUX, *rdb_*LUX, *flc_*BZH and *rdb_*BZH showed a lower presence (3.1%, 3.2%, 3.1% and 6.2%, respectively). The overall presence of admixed individuals in the populations obtained in LUX (6.3%) is about three fold higher than of those obtained in BZH (1.9%), suggesting that outcrossing events have been more frequent in LUX than BZH. Finally, admixed individuals were not detected in any of the original populations, according to the selected threshold [30].

### 2.4. Phenotypic Differentiation

An overall effect of variety was found for all phenotypic traits assessed, reflecting that different varietal types were represented in terms of morpho-phenological and agronomical characteristics. As for the genetic level, differentiation among populations of a given variety was also observed. 

Considering all the assessed traits, days to flowering and leaf length showed the most relevant differentiation, i.e., among populations of most varieties.

**Figure 5:**
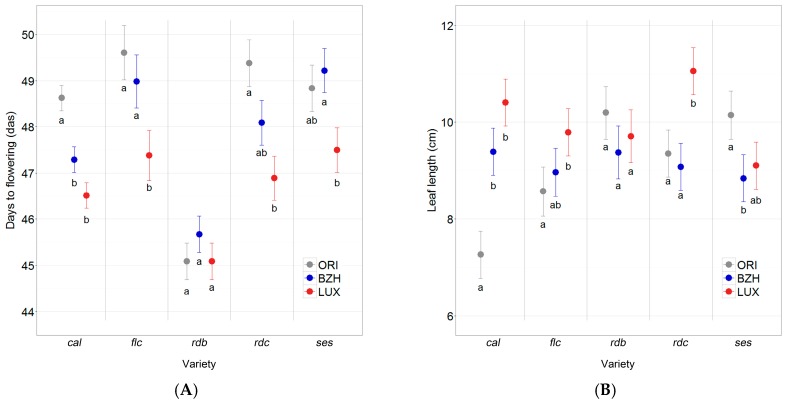
(**A**) Days to flowering in days after sowing (das); and (**B**) Leaf length (cm) of the 15 bean populations belonging to the five studied varieties. Error bars represent standard errors of the means. Within each variety, populations that are not marked with the same lowercase letter differ significantly.

Overall, populations from LUX flowered 1.2 days earlier than populations from BZH (*p* ≤ 0.0001) and 1.6 days earlier than original populations (*p* ≤ 0.0001). When looking into the population effect within each variety, significant differences were found for all varieties but *rdb* (Figure 5A). Phenological differences persisted throughout the season and were confirmed by maturity scores at the end of the growth cycle: populations from LUX obtained significantly higher maturity scores (95% confidence interval of relative effect pd: 0.54–0.60) than populations from BZH (0.44–0.50) and ORI populations (95% confidence interval of relative effect pd: 0.43–0.49). Within individual varieties, this difference among populations was significant only for *cal* (*p* ≤ 0.01) and *rdc* (*p* ≤ 0.0001). 

For leaf length, a significant effect of population within variety was found in all the studied varieties (*p* ≤ 0.0001) with the only exception of *rdb*. Comparisons among populations within varieties resulted in different trends as shown in Figure 5B. No significant differences were found among the populations of any given variety regarding the number of seeds per plant.

## 3. Discussion

### 3.1. Genetic Diversity

The initial diversity of the historical varieties analysed in this study ranged from low (*ses*_ORI) to rather high (*rdc*_ORI). The control variety cal had the lowest rate of initial intra-population diversity, which is in accordance with the uniformity and stability criteria imposed for the registration of modern varieties. Regardless of initial diversity and with the exception of variety *ses*, diversity increased when varieties were displaced from their initial farm site. This observation is particularly relevant for *rdc*, as halo bacterial blight (HBB) had strongly affected this susceptible variety in LUX. In fact, despite the loss of a high number of plants of *rdc* in LUX due to HBB, no genetic bottleneck effect was observed. 

In our experiment, varieties *rdb* and *ses* were multiplied in the farm environment (LUX) which had provided the initial populations. Indeed, the two varieties had been multiplied on the participating farm in LUX for more than ten years before the experiment was set up. For both *rdb* and *ses*, diversity slightly decreased in LUX over the three years of the experiment. 

In summary, according to our data diversity within populations rose when confronted with novel growing conditions in the short term, but decreased in populations multiplied in the same growing environment over more than ten years suggesting a strong role of pedo-climatic conditions on the organization and evolution of populations’ diversity. 

However, we could not confirm this trend due to the lack of the reciprocal populations of varieties *flc* and *rdc* multiplied in the environment from which they were initially provided for the experiment. The evolution of genetic diversity within bean populations as they are transferred across growing environments within networks of gardens and farms constitutes a future research perspective. Nevertheless, the analysis of genetic structure discussed below (Section 3.3) points to some underlying mechanisms.

### 3.2. Genetic Differentiation

As shown by the PCoA and pairwise populations’ F_ST_ results, for varieties *flc* and *rdc*, the populations multiplied in LUX moderately differed both from original (*flc_ORI* and *rdc_ORI*) and from the respective populations multiplied in BZH. Both these populations were initially provided by a seed grower from Aquitaine (AQU) in the southwest of France, where weather conditions are considerably warmer and dryer than in LUX. The significant differentiation of the *flc* and *rdc* populations in LUX may thus be explained by high selection pressure on these populations in the novel growing environment. As discussed above (Section 3.1), the selection pressure in LUX did not lead to a reduction of genetic diversity within LUX populations. 

The differentiation of populations managed by farmers has been evidenced by studies on landraces of common bean [25] and bread wheat [15], which are both mainly autogamous crops. The present experiment demonstrates that the genetic makeup of common bean populations can be influenced by the growing environment within as little as three growing seasons. The growing environment includes natural, as well as conscious selection. Whereas conscious selection was limited to the sorting of bean seeds in this experiment, genetic differentiation may be even more pronounced when plants undergo conscious selection in the fields, as indicated by a former study on variety *flc* [26]. 

### 3.3. Genetic Relationships

Theoretical considerations by Allard [31] provide a clear and useful description regarding the short-term local adaptation of predominantly autogamous species such as common bean at the genetic level. Allard suggests considering the entire populational genotype as “a sort of giant supergene”, because adaptation does not only occur independently at the level of individual loci, but also through highly structured genotypic frequency distributions at the population level. Thereby, the frequency of genotypes conferring high fitness in a given environment is increased in the plant population. At the same time, free genetic variability and recombinational potential within inbreeding populations remain substantial and allow for a response to natural selection in new or changing environments. Indeed, Allard expected mainly autogamous species to accelerate the recombination of genetic information through increased outcrossing in stressful environments. The idea that outcrossing may be induced by the plants’ environment is in accordance with reports of highly variable outcrossing rates of common bean according to environmental conditions [32]. In a study on the generation and maintenance of variability in common bean landraces in Malawi, natural outcrossing was found to be an important factor [33].

This study indicates that such outcrossing may even play a role for the generation and maintenance of variability, as well as local adaptation, when farmers negatively select for seed off-types, as the population genetic structure analysis indicates that the genetic differentiation of populations within bean varieties was due to shifts in allele frequencies and higher occurrence of natural outcrossing events. For varieties *flc* and *rdc* in particular, presence of admixed individuals was highest in the populations multiplied in LUX, the site that presented most severe growing environmental conditions for common bean. In contrast, all three populations of *rdb*—initial and multiplied—showed a certain level of admixture, whereas no admixing appeared for any of the *ses* populations. Based on the theoretical considerations of Allard, one possible explanation for this observation is that the growing conditions in LUX represented a novel, stressful environment for *flc* and *rdc* and favored outcrossing. However, further research would be needed to confirm this hypothesis, also considering that the studied varieties could be characterized by different percentages of outcrossing (Table S2).

### 3.4. Phenotypic Differentiation Indicates Local Adaptation

Phenotypic traits reflect the genetic differentiation of populations and concur to confirm that bean populations can adapt to local growing conditions within as little as three growing seasons. In the jargon of many farmers involved in the in situ maintenance of crop diversity in France and Belgium, a crop population adapted to a given farm environment is called a *lineage* (“souche” in French). Indeed, practitioners recognize different lineages of a given variety by differences at the phenotypic level, rather than the genetic level.

Overall, populations multiplied in LUX had a tendency to flower earlier and develop longer leaves. Both “days to flowering” [25,34,35] and leaf size [36,37] are considered as indicators of local adaptation. Earlier flowering and earlier maturity in populations multiplied in LUX corresponds with a shorter growing season. Longer leaves are in accordance with higher precipitation rates and humidity in LUX, as larger leaves have been identified as an adaptive trait in wet environments [38]. As the numbers of seeds produced per plant did not differ significantly among the populations of any given variety, the mentioned adaptive traits did not apparently affect productivity. Moreover, it is noteworthy that the initial seed lot of the control variety *cal* was provided by the breeding company with an unknown seed treatment (colorless and not declared) of which we became aware only after the experiment was established. Comparisons of this population with the *cal* populations multiplied in the framework of the experiment and which did not undergo any seed treatment must thus be interpreted with much care.

Phenotypic differences among populations cannot be explained by genetics alone. Environmentally induced parental effects, or epigenetic effects, such as physical, chemical and biological seed properties can also influence plant phenotypes and can in some cases be adaptive [39,40,41,42]. Concerning biological seed properties, the assessment of microbial communities associated with bean seeds within the same field experiment showed that these microbial assemblages, in particular their fungal component, were influenced by the seed crops’ environments within two years of multiplication [43]. Although there is increasing evidence for the interdependence of plants and their microbial communities, especially microbial life associated to plant roots [44,45,46], the effect of microbial communities of seeds on plant phenotypes is not well-known [43,47]. 

### 3.5. Implications for the In Situ Management of Bean Diversity in Fields and Gardens

Results of this study, like that of similar studies on other crop species [15,27,28,34,35], enhance our understanding of in situ crop diversity in fields and gardens and alloe to derive conclusions on the management of crop diversity by farmers’ and gardeners’ networks.

The first conclusion concerns the commonly employed term of “in situ conservation”. This study demonstrates that both the genetic and the phenotypic properties of bean populations may be reconfigured as they circulate in a network of seed growers, which manage and use bean diversity dynamically. Thomas [48] has observed that even within a given farm, changing environmental conditions from one growing season to the next drive the dynamic genetic evolution of bread wheat populations managed by farmers, rather than leading to a stable equilibrium. Similar results were reported by Raggi and collaborators for a barley Composite Cross Population having evolved under low-input conditions for 13 years [24]. Our results confirm that common bean diversity managed in fields and gardens constantly evolves in space (as populations are transferred from one grower to another), as well as in time (as populations reproduced in one location evolve with the environmental conditions from one growing season to the next). In such a context, the term “conservation” can be misleading, because it appears as something static rather than dynamic. This is why, in this paper, we have preferred the term “in situ management” of crop diversity.

The second conclusion we draw concerns the role of local adaptation in networks of seed growers managing crop diversity. Since one of the main objectives of in situ management of heterogeneous populations, like landraces and historical varieties, is to maintain diversity [49]—be it for people who wish to cultivate such diverse crop populations or those who want to breed new varieties from old ones—local adaptation is both a means and an aim. It is an aim per se because it can lead to different combinations of morpho-phenological and agronomical traits when whole populations are considered. This aspect may be extremely relevant in view of current uncertainties concerning environmental and climate changes [50] but also considering the transformation of agricultural and food systems [12,13]. For example, in this experiment, local adaptation of bean populations significantly affected flowering time, a trait recognized as adaptive both to cooler climates and drought conditions [51]. In addition, as suggested by different authors [52,53], heterogeneous populations could successfully be used to increase crops’ performance stability by means of intra-specific diversity, especially under organic management conditions.

Concomitantly, local adaptation may also be a means to maintain maximum genetic diversity of a given variety, under the condition that crop diversity management is considered at the collective scale of the farmers’ and gardeners’ network (i.e., the metapopulation), rather than the scale of the individual farm or garden [48,51]. A relevant role of in situ management of crop diversity, in comparison to ex situ, has been shown by several studies on different crops, such as maize [54,55], rice [56] as well as common bean [49,57]. Indeed, the maintenance of several populations of a given variety in contrasted environments within a network constitutes not only a security for in situ crop diversity management, but also a matrix for developing genetic diversity in the form of *lineages* (refer to Section 3.4). In this dynamic process, the phenotype approach of farmers and gardeners is important if variety types are to be maintained. Again, many seed growers select for both plant and seed types to ensure that their *lineage* remains within the respective varietal type (among other selection criteria). Results from this study indicate that selection for varietal type, at least at the basis of seeds, does not prevent outcrossing events from generating and maintaining genetic variability within populations. 

Finally, the dynamic character of both genetic and phenotypic bean diversity revealed by this study points to the role that moving plant populations from one location to another may play for the in situ management of crop diversity. Considering this management as a dynamic process opens new research perspectives, concerning the spatial dynamics of farmers’ and gardeners’ seed networks in particular. 

## 4. Materials and Methods 

In the objective of assessing the short-term adaptation of bean populations to local growing environments under conditions of on-farm multiplication and maintenance of historical varieties, four historical bean varieties and a “modern” control variety were each multiplied on two organic farms with contrasting environmental conditions over three growing seasons (2012–2014). The fourth year (2015), the resulting bean populations were grown in a common garden [58,59]. Phenotypic traits of populations of a given variety were compared and SSR markers assessed. 

### 4.1. Experimental Setup

#### 4.1.1. Varieties

The varieties assessed in the trial are all bush beans with a determinate growth habit. Four historical varieties were selected among those produced on a regular basis by two small-scale, artisanal seed producers participating in the research project. Two varieties originating from a seed grower in the Aquitaine region in southwestern France (AQU) were included in the trial, *Flageolet Chevrier* (*flc*) and *Rognon de Coq* (*rdc*). Two others were provided by a producer in Luxembourg (LUX), *Roi des Belges* (*rdb*) and *St. Esprit à œil rouge* (*ses*). The oldest (*ses*) was first mentioned by Vilmorin-Andrieux in 1855 [60]. The most recent variety (*rdb*) was bred in Belgium in the first half of the 20th century [61]. They were selected for the trial to represent a range of uses (green and shelling beans), earliness and tolerance to diseases, according to the producers’ experience. In addition, seed of the variety *Calima* (*cal*) was obtained from a large-scale breeding company as a modern control. Seed of this variety had been produced in East Africa.

#### 4.1.2. Experimental Design

**Figure 6:**
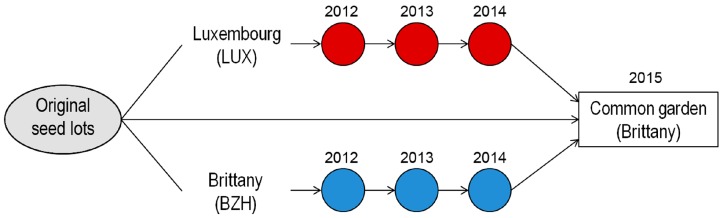
Schematic representation of the multiplication stages carried out for the development of the different bean populations assessed.

Each of the five varieties was multiplied on two organic farms during three years, from 2012 to 2014 (Figure 6). Each year, the preceding year’s harvest was sown on each site. Crops were managed according to the respective farmer’s usual practices (Table S3), including for the sowing date. As the farmers hosting the experiment grow beans on a regular basis, they continued growing other bean varieties on nearby plots, allowing for a potential gene flow between the experimental and non-experimental plots. The farms were located in Brittany, Western France (BZH) and in LUX. The latter is also the farm having contributed the initial seed lots for two of the varieties (*rdb* and *ses*). Each year, the total plot surface per variety ranged from 8.4 to 12.0 m^2^, with an average of 11 m^2^. A description of the experimental sites (BZH and LUX), as well as the farms from which original seed lots originated (AQU and LUX), is given in Supplementary Table S3.

As our objective was to assess the dynamics and local adaptation of bean populations as they are managed by farmers’ and gardeners’ networks, conscious selection was applied to seed lots between each growing season. Seeds that were mechanically damaged, off-type or showing disease symptoms were removed. No conscious selection was applied to bean plants in the experimental plots, based on the observation that farmers involved in seed saving generally sort their seeds, whereas selection in the field is not a generalized practice.

Three years of on-farm multiplication thus resulted in three populations of each variety: the original seed lot used to establish the trials (ORI), and the populations multiplied in BZH and LUX, respectively. Mid-June 2015, all populations were sown in a common garden [58,59] in BZH in the aim of comparing the three populations of each variety. The comparison was designed as split-plot (main plot = variety) in three field replications. Each sub-plot was 2.5 m long and consisted of two rows spaced 75 cm apart. Within rows, plants were spaced at 25 cm to facilitate the observation of individual plants. The farm in BZH was selected for the final trial year, because it was most favorable for bean cultivation. Agronomical, morphological and phenological traits were observed throughout the season and leaf tissue collected for SSR analyses at 68 days after sowing (das).

#### 4.1.3. Sampling for DNA Extraction and Phenotypic Observations

In 2015, twelve bean plants were identified in each subplot for phenotypic observations throughout the growing season, i.e., a total of 36 plants per population. Among them, 30 plants were randomly sampled for DNA extraction in the initial populations (ORI) and 32 in the BZH and LUX populations. Leaf tissues were sampled at flowering.

### 4.2. Plant DNA Extraction and SSR Genotyping

Genomic DNA extraction was performed using the TissueLyser II (Qiagen, Hiden, Germany) and the DNeasy 96 plant kit (Qiagen) on a total of 470 samples according to the procedure provided by the manufacturer. DNA quality and concentration were assessed through UV-Vis spectrophotometry using NanoDrop 2000™ (Thermo Scientific, Waltham, MA, USA).

In order to explore genetic diversity among the initial and the multiplied populations, 35 SSR primer pairs were preliminary tested on a subset of samples (Table S4). Sixteen of them showing a relatively high degree of polymorphism were chosen and, with the aim of analyzing two loci per Linkage Group, 6 other SSR primer pairs (monomorphic on the preliminary tested materials) were included in the analyses for a total of 22 SSR markers. Markers from series AG, BM, GATS [18], BMb [20] and BMd [19,21] were included (Table 3).

In order to reduce time and costs of the molecular analyses, seven multiplex PCR were developed and optimized. Multiplex PCRs were carried out grouping primers with similar annealing temperature and diverse predicted amplicon size. The forward primer of each combination was 5′ end-labelled with one of the following fluorescent dyes: 6-FAM™, VIC^®^, NED™ and ROX™ (Applied Biosystems, Foster City, CA, USA). 

All the reactions were performed using a 2720 Thermal Cycler (Applied Biosystems) in a total volume of 12.5 μL composed as follows: 15 ng of template, 1.25 μL of 10× Buffer (Sigma Aldrich, St. Louis, MO, USA), 1.2 μL of 25 mM MgCl_2_ (Sigma Aldrich), 1.5 μL of 5 mM dNTP mix (Sigma Aldrich), 0.25 μL of 5 U·μL^−1^ Taq DNA Polymerase (Sigma Aldrich) and 0.4 μM of each primer (forward and reverse). The PCR cycle was adapted from Raggi and co-workers [62] and consisted of the following steps: 94 °C for 4 min, followed by 12 cycles of 94 °C for 30 s, *x* °C for 30 s, 72 °C for 30 s, 25 cycles of 94 °C for 10 s, *x* − 1 °C for 15 s, 72 °C for 20 s and 20 min at 72 °C for the final extension, where *x* is the annealing temperature (Table S5). 

Amplicons were denatured using highly deionized formamide and they were subsequently separated an sized using the automatic sequencer AB3130xl Genetic Analyzer (Applied Biosystems) according to size standard GeneScan™ LIZ-500™ (Applied Biosystems). All alleles were visualized and manually scored using the GENEMAPPER^®^ software, version 4.0 (Applied Biosystems).

### 4.3. SSR Data Analysis

Only data obtained from consistent and reproducible SSR were considered for any analyses. Mean allelic richness was calculated up to the number of individuals present in each population using the R package ARES from Van Loon et al. [63] and mean accumulation curves of estimated allelic richness were plotted. Total number of polymorphic markers was assessed considering 95% of major allele frequency as criterion. Consequently, allele frequency (Na) and private alleles by populations were determined using GenAlEx 6.5 software [64], and graphically illustrated by Venn diagrams [65]. 

A population pairwise genetic distance matrix was also calculated and subsequently Principal Coordinates Analysis (PCoA) was performed via distance matrix with data standardization using the previously mentioned software. In order to understand the partition of molecular variance, AMOVA was carried out on the total dataset and for each variety respectively, using 1000 permutations. Population pairwise F_ST_ matrix was also calculated to understand both the degree of diversity and the level of evolution [66] of the studied material using Arlequin 3.5 [67].

With the aim of understanding the genetic structure of the material, a Bayesian approach was used to assign membership of each individual (individuals with more than 20% missing loci were not included in the analysis) using both STRUCTURE [68] and InStruct [69]. The number of clusters was initially tested assuming an admixture model for different clusters (*K*) ranging from 1 to 20. For each tested cluster ten runs were carried out and results were based on a 30,000 burn-in period and a Markov Chain Monte Carlo (MCMC) of 60,000 iterations after burn-in. The effective number of clusters (*K*) was subsequently assessed using the approach of Evanno et al. [70], implemented in STRUCTURE HARVESTER program [71].

Accordingly, a new analysis was accomplished using InStruct software [69] which has been optimized for inferring population structure together with population selfing rates. A single run was performed using a 100,000 burn-in period and 200,000 MCMC iterations. The result was visualized with Distruct software [72]. A threshold of *q* > 0.7 was considered to assign an individual to a cluster [30].

### 4.4. Observation of Phenotypic Traits

During the multiplication of populations in BZH and LUX between 2012 and 2014, plants were assessed for morpho-phenological and agronomical traits, as well as scored for plant health traits. In the common garden in 2015, the flowering date was noted for each plant as the date of the first open flower. In addition, maturity was scored at 95 das with a scale from 1 (all pods are green) to 3 (most pods dry, harvest maturity). Concerning morphology, the length of the middle leaflet of the fully developed third trifoliate leaf was measured at 43 das [73]. The length of stems was measured at flowering growth stage [74]. 

Throughout crop development, disease symptoms (leaf mosaic, leaf blustering, blight spots and phloem necrosis) were scored on a scale from 1 (no symptom) through 3 (medium symptom expression) to 5 (very strong symptoms, plant dying). At flowering and mid seed fill growth stages, overall plant vigor was scored on a scale from 1 (very little vigor) to 5 (very vigorous). Finally, after harvest, the number of pods, empty pods and seeds produced per plant were counted.

In the common garden in 2015, all populations of variety *flc* were all but completely lost to Necrotic Black Root Syndrome—a systemic phloem necrosis [75]—leading to missing data for phenotypic traits observed after flowering.

### 4.5. Analysis of Phenotypic Data

For phenotypic traits, statistics were computed using the programming language and software environment R version 3.3.0 [76]. In tests, null hypotheses with *p*-values below the significance level (α) of 0.05 were rejected.

Interval and ratio type data (days to flowering, leaf length, stem length, 1000-seed weight, number of seeds per plant) were analyzed with linear mixed effects models using the R package “nlme” [77,78]. Nesting (main and sub-plots) and pseudo-replication (several plants observed per subplot) were taken into account in the model by setting appropriate random effects. Firstly, the overall effect of variety, version and variety*version interaction was tested in a model including the data over all varieties, where “version” stands for the origin of the population (that is: BZH, LUX or ORI) [34]. Secondly, the effect of version was specified within each variety by subsetting the data and building the linear mixed effects model with only version as fixed effect. Least square means were computed with the package “lsmeans” [79], as well as Tukey’s Honestly Significant Difference (HSD) test for multiple comparisons.

For ordinal variables (score data), rank-based ANOVA-type statistic [80,81] was computed by the “rankFD” [82] R package. Nesting and pseudo-replication were taken into account by calculating ANOVA-type statistics in two steps. Relative effects (pd) were computed for appropriate two-way comparisons.

### 4.6. Semi-Directive Interviews

Between August 2014 and June 2016, a total of 21 people were interviewed in the framework of a qualitative sociological study on the management of bean diversity and plant health. The thematic guide used for the interviews is presented in Supplementary Table S6. Fifteen interviews were conducted, with up to 3 people per interview, resulting in approximately 300 pages of interview transcription in total. The outcomes of this study are yet to be published, but have contributed to better understanding and situating the results of the experimentation, thereby contributing to their discussion.

## 5. Conclusions

As a final conclusion, this study has shown the ability of common bean historical varieties to adapt, both genetically and phenotypically, to local growing conditions within as little as three growing seasons. When considering farmers’ and gardeners’ networks engaged in the management of bean diversity at the collective scale, processes of local adaptation may contribute to maintaining genetic and phenotypic diversity at the level of the metapopulation. Concomitantly, this diversity constitutes a resource upon which to meet the needs of food systems and environmental challenges in the future. In view of supporting the efforts of actors engaged with in situ crop biodiversity management and conservation, our findings are an invitation to have a closer look at the dynamics within crop diversity networks, in particular the circulation of seed lots among different growers and the effect it may have on genetic and phenotypic diversity.

## Figures and Tables

**Table 1:** Different populations of historical and modern bean varieties developed and assessed in the experiment. The 1000-seed weight (TSW) of each seed lot is also reported.

Variety	Site/Origin	Population (Code)	TSW (g)	Information on Original Seed Lot
*Calima* (*cal*) modern variety (control)	Original seed lot	*cal_ORI*	250	Harvested in East Africa in 2010
	Brittany	*cal_BZH*	278	
	Luxembourg	*cal_LUX*	301	
*Flageolet Chevrier* (*flc*) historical variety	Original seed lot	*flc_ORI*	235	Harvested in AQU in 2011 (organic)
	Brittany	*flc_BZH*	244	
	Luxembourg	*flc_LUX*	256	
*Rognon de Coq* (*rdc*) historical variety	Original seed lot	*rdc_ORI*	340	Harvested in AQU in 2011 (organic)
	Brittany	*rdc_BZH*	415	
	Luxembourg	*rdc_LUX*	452	
*Roi des Belges* (*rdb*) historical variety	Original seed lot	*rdb_ORI*	442	Harvested in LUX in 2011 (organic)
	Brittany	*rdb_BZH*	320	
	Luxembourg	*rdb_LUX*	376	
*St. Esprit à œil rouge* (*ses*) historical variety	Original seed lot	*ses_ORI*	884	Harvested in LUX in 2011 (organic)
	Brittany	*ses_BZH*	682	
	Luxembourg	*ses_LUX*	n.a.	

n.a. = not available.

**Table 2:** Pairwise F_ST_ among populations of five varieties of common bean calculated upon SSR data. ORI stands for “original seed lot”, BZH for “Brittany” and LUX for “Luxembourg”.

Variety	ORI vs. BZH	ORI vs. LUX	BZH vs. LUX
“Flageolet Chevrier” ( *flc*)	0.01	0.12 ***	0.09 ***
“Rognon de Coq” ( *rdc*)	0.03	0.19 ***	0.20 ***
“Roi des Belges” ( *rdb*)	0.01	0.00	0.00
“St. Esprit” ( *ses*)	0.07	0.06	−0.02
“Calima” ( *cal*)	0.02	0.01	0.03

*** *p* ≤ 0.001.

**Table 3:** List of tested primer pairs.

Marker	Linkage Group	SSR Motif	Primer FOR	Primer REV	Predicted Size	References
BM200	b01	(AG)_10_	TGGTGGTTGTTATGGGAGAAG	ATTTGTCTCTGTCTATTCCTTCCAC	221	[ 19]
BM140	b04	(GA)_30_	TGCACAACACACATTTAGTGAC	CCTACCAAGATTGATTTATGGG	190	[ 19]
BMb43	b04	(TA)_10_	GTGATCGGCTACATTAGCAT	GCTCTCATGTTCTCTTTCTCA	143	[ 20]
BM175	b05	(AT)_5_-(GA)_19_	CAACAGTTAAAGGTCGTCAAATT	CCACTCTTAGCATCAACTGGA	170	[ 19]
BMb293	b05	(CTT)_7_	CAATTCTACACTTTGGTGGG	AACGTCATTGATTTGACTCC	154	[ 20]
BM137	b06	(CT)_33_	CGCTTACTCACTGTACGCACG	CCGTATCCGAGCACCGTAAC	155	[ 19]
BM170	b06	(CT)_5_-(CT)_12_	AGCCAGGTGCAAGACCTTAG	AGATAGGGAGCTGGTGGTAGC	179	[ 18]
BMb526	b07	(TA)_15_	AAAGGGCAAGTTAGATGTGA	TTTGAAGAATAGAAATCATACTG	220	[ 20]
BM201	b07	(GA)_15_	TGGTGCTACAGACTTGATGG	TGTCACCTCTCTCCTCCAAT	102	[ 19]
BM189	b08	(CT)_13_	CTCCCACTCTCACCCTCACT	GCGCCAAGTGAAACTAAGTAGA	114	[ 19]
BMd-44	b08	(AG)_5_	GGCAGCTTACTAACCCGAAA	TTCCTTCCCCTTTCTTCTCC	135	[ 21]
BM141	b09	(GA)_29_	TGAGGAGGAACAATGGTGGC	CTCACAAACCACAACGCACC	218	[ 19]
BM114	b09	(TA)_8_(GT)_10_	AGCCTGGTGAAATGCTCATAG	CATGCTTGTTGCCTAACTCTCT	234	[ 18]
BM157	b10	(GA)_16_	ACTTAACAAGGAATAGCCACACA	GTTAATTGTTTCCAATATCAACCTG	113	[ 18]
BMb356	b01	(TA)_14_	TCCGAATTTCTTAATTTCACTT	ATCGCGGATTTATATGTGTC	187	[ 20]
BMb221	b10	(AT)_10_	TGAAAGACAAGAGGGTTCAT	TTGTAGGCACTATTCCGTTT	223	[ 20]
BMd-41	b11	(ATT)_9_	CAGTAAATATTGGCGTGGATGA	TGAAAGTGCAGAGTGGTGGA	250	[ 21]
BMb619	b11	(AT)_22_	GATGGACACACTCACAAACA	TGTGTTCTACCACCAACAGA	298	[ 20]
GATS91	b02	(GA)_11_	GAGTGCGGAAGCGAGTAGAG	TCCGTGTTCCTCTGTCTGTG	229	[ 19]
BM156	b02	(CT)_32_	CTTGTTCCACCTCCCATCATAGC	TGCTTGCATCTCAGCCAGAATC	267	[ 19]
AG01	b03	(GA)_8_-(GA)_5_-(AG)_4_	CATGCAGAGGAAGCAGAGTG	GAGCGTCGTCGTTTCGAT	132	[ 19]
BM172	b03	(GA)_23_	CTGTAGCTCAAACAGGGCACT	GCAATACCGCCATGAGAGAT	107	[ 19]

## Supplementary Materials

Supplementary materials can be found at www.mdpi.com/1422-0067/18/3/493/s1.

## Abbreviations

MDPI	Multidisciplinary Digital Publishing Institute
SSR	Simple sequence repeat/microsatellite
TSW	1000-seed weight
*cal*	‘Calima’
*flc*	‘Flageolet Chevrier’
*rdb*	‘Roi des Belges’
*rdc*	‘Rognon de Coq’
*ses*	‘Saint Esprit à œil rouge’
AQU	Aquitaine
BZH	Brittany
LUX	Luxembourg
ORI	Original seed lot employed to establish the field trial
AMOVA	Analysis of Molecular Variance
*He*	Expected heterozygosity
PCoA	Principal coordinate analysis
F_ST_	Fixation index
das	Days after sowing
HBB	Halo bacterial blight
Na	Number of alleles
MCMC	Markov Chain Monte Carlo
HSD	Tukey’s Honestly Significant Difference
pd	Relative effect
