# Supplementary material for: Short-Term Local Adaptation of Historical Common Bean (Phaseolus vulgaris L.) Varieties and Implications for In Situ Management of Bean Diversity
Source: Int J Mol Sci. 2017 Feb 28;18(3):493. doi: 10.3390/ijms18030493 (PMC5372509; doi:10.3390/ijms18030493)
Supplement: Supplementary file 1 [file ijms-18-00493-s001.zip › ijms-172941-Supplementary Tables.pdf]

Table S1. AMOVA on three populations of the each bean variety

Variety	Source of variation	d.f.	Sum of squares	Variance of components	Percentage of variation
flc	Among populations	2	10 747	0.07312	8.44***
	Within populations	185	146 758	0.79329	91.56
	Total	187	157 505	0.86641	
rdc	Among populations	2	45 623	0.333	14.34***
	Within populations	185	367 441	1 987	85.66
	Total	187	413 064	2 319	
rdb	Among populations	2	2 185	0.00217	0.23
	Within populations	185	177 001	0.95676	99.77
	Total	187		0.95893	
ses	Among populations	2	0.381	0.00232	4.89*
	Within populations	185	8 353	0.04515	95.11
	Total	187	8 734	0.04747	
cal	Among populations	2	0.693	0.003	2.28
	Within populations	185	26 025	0.141	97.72
	Total	187	26 718	0.144	

*** *p≤ 0.001*

* p≤ 0.05

Table S2. Posterior population selfing rates as inferred by InStruct software

	Mean	Var
Cluster 1 (flc)	0.868	0.002
Cluster 2 (rdc)	0.886	0.002
Cluster 3 (ses)	0.911	0
Cluster 4 (cal)	0.912	0
Cluster 5 (rdb)	0.928	0

Table S3. Weather and soil conditions, as well as management practices, at the two experimental sites (BZH, LUX), and sites having provided initial seed lots (AQU, LUX)

	**Aquitaine (AQU)**	**Luxembourg (LUX)**	**Brittany (BZH)**
Soil and climatic conditions			
Latitude	44°21'13.32''N	49° 42' 7.42''N	48°2'57.50''
Longitude	0°31'31.06''E	6° 2' 20.43''E	1°47'10.96''
Altitude (m above sea level)	88	259	34
Average annual:			
- minimum temperature (°C)	8.6	5.2	7.7
- maximum temperature (°C)	19.1	15.0	16.7
- average temperature (°C)	13.4	9.6	11.9
- rainfall (mm)	644	788	694
Soil type	Silty clay	Sandy clay	Clay-loam
Soil pH	8.5	8.1	6.4
Organic matter content	1.9	2.7	2.6
Mineral nitrogen (kg N / ha)	11.1	29.6	102.9
Phosphorous (mg P_2O_5 / kg)	36	15	144
Potassium (mg K_2O / kg)	140	141	385
Calcium (mg CaO / kg)	12380	8848	1644
Magnesium (mg MgO /kg)	261	110	188
Crop management			
Soil preparation	Subsoiler every 3-4 years (60 cm)<br>Plough (18 cm)<br>'Actisol' harrow (10 cm)<br>Rotary cultivator	Rotary tiller (maximum 20 cm)	Rotary cultivator (15 cm)<br>Shank cultivator (30 cm)<br>Spring-tooth harrow (15 cm)
Distance between rows	60 cm	75 cm	75 cm
Distance between plants	2012: 10 cm<br>2014: approx. 5 cm	2012-13: 10 cm<br>2014: approx. 5 cm	2012-13: 10 cm 10 cm<br>2014: approx. 5 cm
Irrigation	overhead; to field capacity every 10 days in absence of rain	overhead;<br>to field capacity	none
Fertilization	Composted farm yard manure every 3-4 years (75 t/ha), followed by green manure	In crop rotation:<br>Green manure, compost<br>Application of on-farm preparation of "effective microorganisms"	none

Table S4. List of 35 SSR markers initially tested

MARKER	ID	LINKAGE GROUP	SSR MOTIF	PRIMER FOR	PRIMER REV	PREDICTED SIZE	REFERENCES
BM200	SSR1	b01	$(AG)_{10}$	TGGTGGTTGTTATGGGAGAAG	ATTTGTCTCTGTCTATTCCTTCCAC	221	Blair et al. [20]
BMb64	SSR2	b01	$(TA)_{21}$	GGTGTTGGCGATATAAAGTC	TATAATGGAACCCATAACGG	231	Córdoba et al. [21]
BMb356	SSR3	b01	$(TA)_{14}$	TCCGAATTTCTTAATTTCACTT	ATCGCGGATTTATATGTGTC	187	Córdoba et al. [21]
BM139	SSR4	b02	$(CT)_{25}$	TTAGCAATACCGCCATGAGAG	ACTGTAGCTCAAACAGGGCAC	115	Blair et al. [20]
GATS91	SSR5	b02	$(GA)_{11}$	GAGTGCGGAAGCGAGTAGAG	TCCGTGTTCCTCTGTCTGTG	229	Blair et al. [20]
BM156	SSR6	b02	$(CT)_{32}$	CTTGTTCCACCTCCCATCATAGC	TGCTTGCATCTCAGCCAGAATC	267	Blair et al. [20]
AG01	SSR7	b03	$(GA)_{8}$-$(GA)_{5}$-$(AG)_{4}$	CATGCAGAGGAAGCAGAGTG	GAGCGTCGTCGTTTCGAT	132	Blair et al. [20]
GATS54	SSR8	b03	$(GA)_{5}$-$(GA)_{8}$	GAACCTGCAAAGCAAAGAGC	TCACTCTCCAACCAGATCGAA	114	Blair et al. [20]
BM172	SSR9	b03	$(GA)_{23}$	CTGTAGCTCAAACAGGGCACT	GCAATACCGCCATGAGAGAT	107	Blair et al. [20]
BM140	SSR10	b04	$(GA)_{30}$	TGCACAACACACATTTAGTGAC	CCTACCAAGATTGATTTATGGG	190	Blair et al. [20]
BMb43	SSR11	b04	$(TA)_{10}$	GTGATCGGCTACATTAGCAT	GCTCTCATGTTCTCTTTCTCA	143	Córdoba et al. [21]
BMb488	SSR12	b04	$(AT)_{16}$	TTGCTTATTGTTTCCGATTT	AAGCCTTGCAAAGAGTTAAA	236	Blair et al. [20]
BM175	SSR13	b05	$(AT)_{5}$-$(GA)_{19}$	CAACAGTTAAAGGTCGTCAAATT	CCACTCTTAGCATCAACTGGA	170	Blair et al. [20]
BMb293	SSR14	b05	$(CTT)_{7}$	CAATTCTACACTTTGGTGGG	AACGTCATTGATTTGACTCC	154	Córdoba et al. [21]
BMb560	SSR15	b05	$(AT)_{13}$	AACTCATGAGGTGAGGTTTG	GAGGAGGAGGGAATCTATTG	265	Córdoba et al. [21]
BM137	SSR16	b06	$(CT)_{33}$	CGCTTACTCACTGTACGCACG	CCGTATCCGAGCACCGTAAC	155	Blair et al. [20]
BM170	SSR17	b06	$(CT)_{5}$-$(CT)_{12}$	AGCCAGGTGCAAGACCTTAG	AGATAGGGAGCTGGTGGTAGC	179	Gaitàn-Solìs et al. [19]
BMd-12	SSR18	b06	$(AGC)_{7}$	CATCAACAAGGACAGCCTCA	GCAGCTGGCGGGTAAAACAG	167	Blair et al. [22]
BM160	SSR19	b07	$(GA)_{15}$-$(GAA)_{5}$	CGTGCTTGGCGAATAGCTTTG	CGCGGTTCTGATCGTGACTTC	211	Blair et al. [20]
BMb526	SSR20	b07	$(TA)_{15}$	AAAGGGCAAGTTAGATGTGA	TTTGAAGAATAGAAATCATACTG	220	Córdoba et al. [21]
BM201	SSR21	b07	$(GA)_{15}$	TGGTGCTACAGACTTGATGG	TGTCACCTCTCTCCTCCAAT	102	Blair et al. [20]
BM189	SSR22	b08	$(CT)_{13}$	CTCCCACTCTCACCCTCACT	GCGCCAAGTGAAACTAAGTAGA	114	Blair et al. [20]
BMb445	SSR23	b08	$(TA)_{9}$	CCAAGCTCTGAATCAATCAT	CCAAGTTAACAAATTGAGCC	151	Córdoba et al. [21]
BMd-44	SSR24	b08	$(AG)_{5}$	GGCAGCTTACTAACCCGAAA	TTCCTTCCCCTTTCTTCTCC	135	Blair et al. [22]
BMb266	SSR25	b08	$(TAA)_{6}$	AAATTCAAACCAGCCATTC	GGCAATTACATTTGGAGAAA	156	Córdoba et al. [21]
BM141	SSR26	b09	$(GA)_{29}$	TGAGGAGGAACAATGGTGGC	CTCACAAACCACAACGCACC	218	Blair et al. [20]
BM114	SSR27	b09	$(TA)_{8}(GT)_{10}$	AGCCTGGTGAAATGCTCATAG	CATGCTTGTTGCCTAACTCTCT	234	Gaitàn-Solìs et al. [19]
BM188	SSR28	b09/B11	$(CA)_{18}(TA)_{7}$	TCGCCTTGAAACTTCTTGTATC	CCCTTCCAGTTAAATCAGTCG	153/200	Blair et al. [20]
BM157	SSR29	b10	$(GA)_{16}$	ACTTAACAAGGAATAGCCACACA	GTTAATTGTTTCCAATATCAACCTG	113	Gaitàn-Solìs et al. [19]
BM221	SSR30	b10	$(AT)_{10}$	TGAAAGACAAGAGGGTTCAT	TTGTAGGCACTATTCCGTTT	223	Córdoba et al. [21]
BMb96	SSR31	b10	$(CA)_{11}$	CATAAAGCACGTCACTTCAA	GCCTTGGACACTACCATTT	126	Córdoba et al. [21]
BMd-41	SSR32	b11	$(ATT)_{9}$	CAGTAAATATTGGCGTGGATGA	TGAAAGTGCAGAGTGGTGGA	250	Blair et al. [22]
BMb32	SSR33	b11	$(AT)_{22}$	CTGACCTCGATCTTTCTGAG	GAACCATCCAGTTAAACCAA	253	Córdoba et al. [21]
BMb10	SSR34	b11	$(AT)_{14}$	GAGGCAATTCGTTTGAAATA	AGACAATCCGTTATACAATCT	225	Córdoba et al. [21]
BMb619	SSR35	b11	$(AT)_{22}$	GATGGACACACTCACAAACA	TGTGTTCTACCACCAACAGA	298	Córdoba et al. [21]

Table S5. Durations and temperatures of PCR cycles employed for SSR markers

MARKER	PRIMER	Tm (°C)	Ta (°C)	MARKER	PRIMER	Tm (°C)	Ta (°C)
BM140	For	61.2	56	BM141	For	67.4	59
	Rev	61			Rev	66.6	
BM175	For	60.8	56	BM114	For	63.1	59
	Rev	61.8			Rev	62.3	
BMb43	For	59.2	56	BMD-41	For	63.4	59
	Rev	58.8			Rev	64.7	
BMb221	For	58.7	56	GATS91	For	64.2	59
	Rev	58.7			Rev	64.1	
BMb293	For	59.5	56	BM156	For	67.6	59
	Rev	59			Rev	68.7	
BMb356	For	58.3	56	AG01	For	63.9	59
	Rev	58.9			Rev	64.1	
BMb526	For	58.8	56	BM172	For	63.3	59
	Rev	54.4			Rev	63.9	
BMb619	For	59.6	56	BM157	For	61.4	59
	Rev	59.2			Rev	61.2	
BM200	For	63.6	59	BM137	For	65.7	59
	Rev	62.4			Rev	66.4	
BM189	For	63.4	59	BM170	For	63.3	59
	Rev	62.2			Rev	63.4	
BMd- 44	For	63	59	BM201	For	61.4	59
	Rev	63.3			Rev	61.3	

Tm melting temperature
Ta annealing temperature

Table S6. Thematic guide employed for semi-directive interviews conducted between 2014 and 2016

Research question

How is common bean diversity and health managed by the artisanal seed companies among the Croqueurs de Carottes?

Possible initial questions

Seed growers: *How do you manage seedborne diseases in your production system?*
Researchers: *Please explain your work concerning bean blights.*
Others (institutions…): *What role do you play in the seed sector with respect to seed quality?*

Seed quality

Quality of seed [definition, plant health, variety, criteria, alternatives, expectations]
Ensure quality [practices, cultivation, selection, analyses, tests, inspection, difficulties, customer expectations]

Production systems

Agriculture [peasant / industrial, conventional / organic, relations with society]
Seed production [peasant / industrial, adaptation, varieties, meaning]
Plant diseases [Definition, meaning, disease control, problematization]
Ecosystem [soil, microorganisms, plants, growers]

Plant health legislation

Role of legislation [in general, advantages and disadvantages, necessity]
Impact on production [enforcement, inspection, sanctions, practices]
Actors [legislators, enforcement, institutions, FNAMS, 'PV', inspectors, researchers, NGO, relations]
Difficulties [for practice, according to production system, enforcement, understanding, information]

Competences and practices

Role [profession, engagement, motivation]
Competences [training, experience, curriculum]
Everyday life [relations at work, location, tasks]

Relations

Growers [network, organization, advice, customers]
Institutions ['PV', FNAMS, GNIS, RSP, INRA]
Research [among researchers / disciplines, research and practice]
Politics [political action, network]
