# Supplementary material for: Short-Term Local Adaptation of Historical Common Bean (Phaseolus vulgaris L.) Varieties and Implications for In Situ Management of Bean Diversity
Source: Int J Mol Sci. 2017 Feb 28;18(3):493. doi: 10.3390/ijms18030493 (PMC5372509; doi:10.3390/ijms18030493)
Supplement: Supplementary file 1 [file ijms-18-00493-s001.zip › ijms-172941-Supplementary Figures.pdf]

Supplementary Materials: Short-Term Local Adaptation of Historical Common Bean (*Phaseolus vulgaris* L.) Varieties and Implications for In Situ Management of Bean Diversity

Stephanie M. Klaedtke, Leonardo Caproni, Julia Klauck, Paul de la Grandville, Martin Dutartre, Pierre M. Stassart, Véronique Chable, Valeria Negri [3] and Lorenzo Raggi

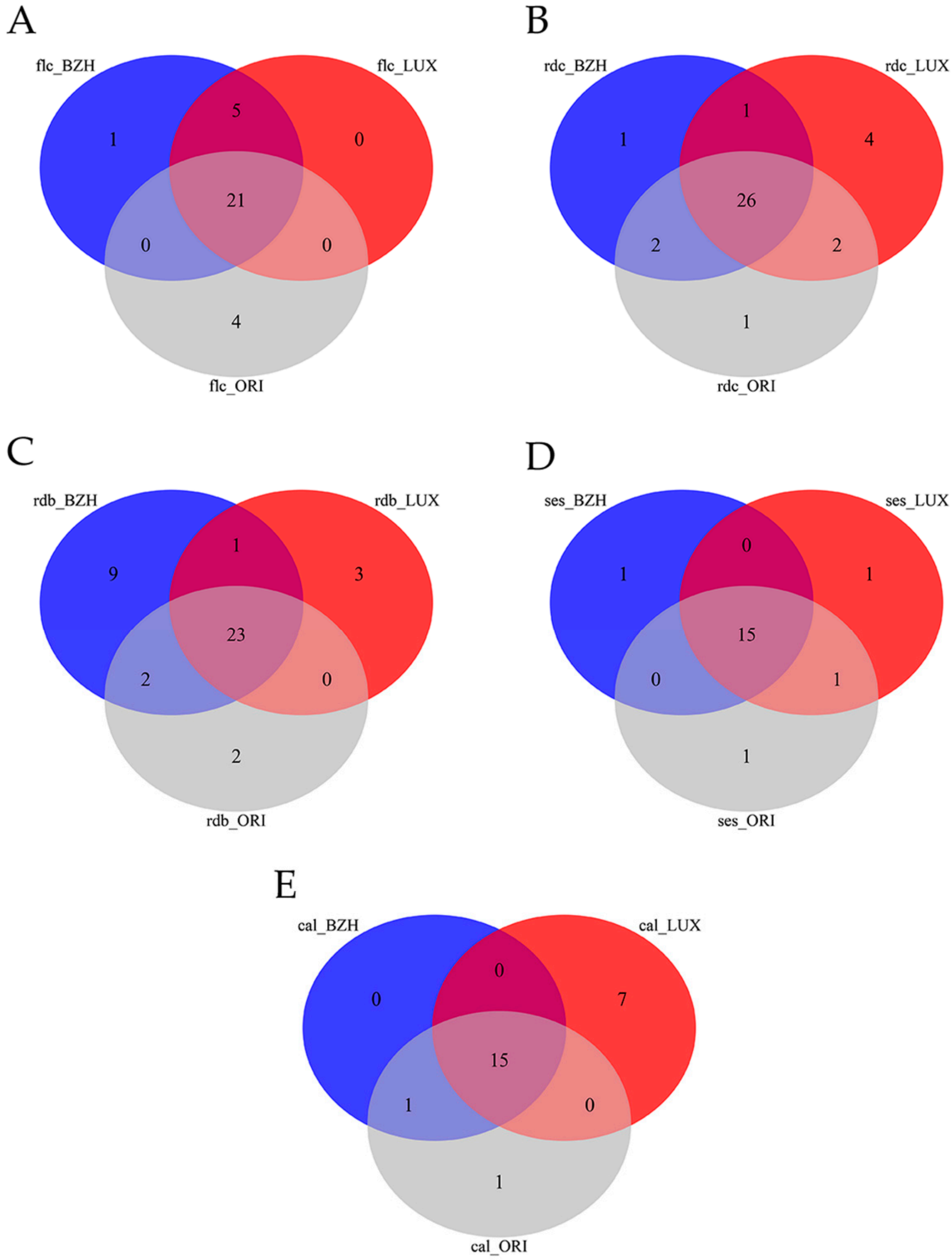

Figure S1. Venn diagrams showing allele distributions among three populations for each bean variety.

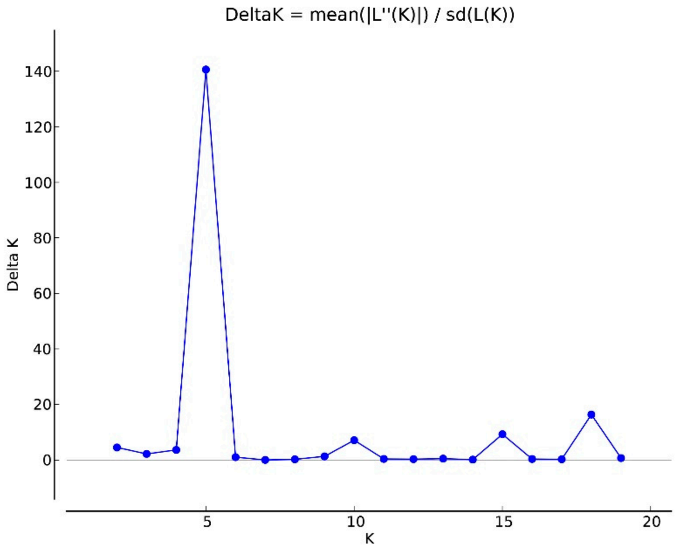

Figure S2. Levels of population subdivision according to STRUCTURE HARVESTER analysis.
